# Clinical evaluation of a novel *H. pylori* fecal molecular diagnosis kit (multiplex RT-PCR method) for detecting clarithromycin and fluoroquinolones resistance using stool samples

**DOI:** 10.3389/fcimb.2025.1592612

**Published:** 2025-06-23

**Authors:** Wen-juan Wei, Bang sun He, Bin Lv, Bin Yang, Yong Xie, Zhen yu Zhang

**Affiliations:** ^1^ Department of Gastroenterology, Nanjing First Hospital, Nanjing Medical University, Nanjing, Jiangsu, China; ^2^ Department of Laboratory Medicine, Nanjing First Hospital, Nanjing Medical University, Nanjing, Jiangsu, China; ^3^ Department of Gastroenterology, The First Affiliated Hospital of Zhejiang Chinese Medical University, Hangzhou, Zhejiang, China; ^4^ Department of Gastroenterology, Jiangsu Taizhou People’s Hospital, Taizhou, Jiangsu, China; ^5^ Jiangxi Clinical Research Center for Gastroenterology, First Affiliated Hospital of Nanchang University, Department of Gastroenterology, Jiangxi Provincial Key Laboratory of Digestive Diseases, Nanchang, China

**Keywords:** *Helicobacter pylori*, fecal diagnostic kit, clarithromycin resistance, fluoroquinolones resistance, molecular diagnosis

## Abstract

**Background:**

*Helicobacter pylori* infection poses a significant global health challenge, exacerbated by rising antibiotic resistance. This study aimed to evaluate a novel multiplex RT-PCR-based fecal diagnostic kit (Cowin Biosciences, Jiangsu, China) for detecting mutations in the *23S rRNA* and *gyrA* genes associated with clarithromycin and fluoroquinolone resistance in *H. pylori*.

**Methods:**

A total of 1,176 participants from four clinical centers in China were enrolled between August 2022 and October 2023. Phenotypic resistance was assessed on *H. pylori* isolated from gastric samples using the minimum inhibitory concentration (MIC) method (E-test), while fecal samples were analyzed molecularly via the diagnostic kit and Sanger sequencing. Positive (PPA), negative (NPA), and overall percentage agreement (OPA) with 95% confidence intervals (CI) were calculated.

**Results:**

There was a high level of consistency between phenotypic testing and novel diagnostic kit. The PPA, NPA, and OPA of the fecal diagnostic kit for detecting clarithromycin susceptibility were 92.97% (CI: 90.6%-95.3%), 87.89% (CI: 85.0%-90.8%), and 90.36% (CI: 88.5%-92.3%), respectively. And likewise, the PPA, NPA, and OPA in diagnosing *H*. *pylori* resistance to fluoroquinolones were 86.85% (CI:83.6%- 90.1%), 91.12% (CI: 88.6%-93.7%), and 89.12% (CI: 87.1%-91.1%), respectively. Beyond that, the diagnostic kit also exhibited a high degree of concordance with the outcomes of Sanger sequencing. Specifically, when assessing clarithromycin resistance, the PPA, NPA, and OPA of the diagnostic kit were 97.13% (CI: 95.7%-98.6%), 94.62% (CI: 92.6%-96.6%), and 95.90% (CI: 94.7%-97.1%), respectively. Similarly, application of the diagnostic kit to detect fluoroquinolone resistance achieved a PPA of 96.92% (CI: 94.9%-98.4%), an NPA of 93.18% (CI: 91.0%-95.3%), and an OPA of 94.69% (CI: 93.3%-96.1%). The concordance rate between the fecal kit and phenotypic susceptibility testing varied with MIC values. For clarithromycin resistance, the positive percentage agreement (PPA) was lowest (89.74%) when MIC exceeded 256 µg/mL but peaked at 96.47% for MIC values between 32 and 256 µg/mL. The highest overall concordance (Kappa = 0.774) was observed at intermediate MIC levels (4–32 µg/mL), suggesting optimal detection accuracy in this range.

**Conclusions:**

The fecal diagnostic kit provides a rapid, non-invasive, and reliable method to predict clarithromycin and fluoroquinolone resistance, supporting personalized therapy.

**Clinical Trial Registration:**

ClinicalTrials.gov, identifier NCT05410652.

## Introduction

*Helicobacter pylori (H. pylori)*, an important human pathogen affecting over 50% of the global population, is etiologically associated with chronic gastritis, peptic ulcers, and gastric malignancies, including its classification as a Group I carcinogen by the World Health Organization (Pastukh et al., 2017). Previous studies have shown that gastric cancer is the third most prevalent malignant tumor with high incidence rates within China (Zhu and Li, 2010). Nonetheless, a recent study by the National Cancer Center of China indicates a decline in the overall prevalence of gastric cancer; the study further reports that gastric cancer in males ranks fourth in the incidence rate of malignant tumors and sixth amongst females (Han et al., 2024). A decrease in gastric cancer rates may be associated with increased awareness of *H. pylori* detection and treatment.

The Chinese Society for *Helicobacter* Research guidelines recommend *H. pylori* eradication treatment as the major preventive method for gastric cancer. In a significant proportion of regions across China however, there is a high resistance rate of *H. pylori* to first-line eradication drugs including clarithromycin and fluoroquinolones. The primary and secondary antibiotic resistance rates of *H. pylori* stand at 37% and 77% for clarithromycin, and 34% and 62% for levofloxacin (Zhong et al., 2021). The conventional antibiotic susceptibility testing typically requires about one week to yield results, making it time-intensive and expensive within clinical practice. Therefore, there is an urgent need for clinicians to adopt a novel, expedited approach to determining antibiotic sensitivity.

Molecular testing is a promising technique for diagnosing *H. pylori* and can identify DNA mutations in bacteria that lead to antibiotic resistance. The Maastricht VI/Florence consensus report highlights molecular methods—specifically real-time PCR, whole-genome sequencing, and digital PCR—for detecting *H. pylori* mutations linked to clarithromycin, levofloxacin, tetracycline, and rifampicin resistance (Malfertheiner et al., 2022). The accuracy of molecular detection techniques for predicting antibiotic resistance has significant variability across different antibiotics (Wang et al., 2018). Many studies have demonstrated that PCR and sequencing-based detection methods can reliably predict resistance to both quinolone drugs and clarithromycin (Wang et al., 2018; Egli et al., 2020).

Various methodologies have been documented for detecting *H. pylori* resistance mutations to clarithromycin and levofloxacin (Fontana et al., 2003; Trespalacios et al., 2015; Giorgio et al., 2016; Beckman et al., 2017; Karmakar et al., 2023). These techniques use diverse sample types, such as gastric mucosa, gastric juice, and feces, for detection and analysis. Pastukh et al. reported that the GenoType HelicoDR kit has a 94.3% positive detection rate for *H. pylori* in gastric mucosa and can identify molecular resistance to clarithromycin and levofloxacin (Pastukh et al., 2017). The GenoType HelicoDR kit (Hain Lifescience, Germany) employs a reverse hybridization assay to detect clarithromycin and fluoroquinolone resistance mutations. DNA extracted from gastric biopsies is amplified via multiplex PCR, followed by hybridization of biotinylated amplicons to membrane-bound probes. Mutations are visualized through colorimetric reactions, enabling rapid phenotypic resistance profiling. In Japan, a novel POCT kit for *H. pylori* and clarithromycin resistance utilizes loop-mediated isothermal amplification (LAMP) targeting *ureA* and *23S rRNA* genes directly from gastric juice. LAMP bypasses thermal cycling, allowing real-time fluorescence detection of resistance mutations within 60 minutes. This approach achieves 100% sensitivity, and 95.9% specificity compared to culture (Tsuda et al., 2022). Elsewhere, another study assessed the efficacy of the LightMix^®^ RT-PCR assay in detecting clarithromycin resistance in gastric biopsy and stool samples. This assay demonstrated a 95% concordance rate with phenotypic clarithromycin resistance screening conducted using the E-Test^®^ (Redondo et al., 2018).

1) The presented study evaluated the performance of a novel fecal *gyrA/23S rRNA* fecal molecular diagnostic kit (developed by Cowin Biosciences, Jiangsu, China), which was subsequently approved by the China’s National Medical Products Administration (NMPA) (Registration No. 20243402348)) and commercialized for clinical use. The kit utilizes fluorescence PCR melting curve analysis to detect *H. pylori* resistance mutations responsible for clarithromycin and fluoroquinolones. Briefly, allele-specific primers and dual-labeled TaqMan-MGB probes (FAM/ROX) were designed to target wild-type and mutant sequences at *23S rRNA* (A2142G/C/A2143G) and *gyrA* (N87K/D91N/Y/G) loci. Through asymmetric PCR amplification single-stranded DNA products were generated for probe hybridization. Post-amplification melt curve analysis differentiated mutant and wild-type alleles based on thermodynamic stability differences. The *gyrA/23S rRNA* fecal molecular diagnostic kit enables clinicians to non-invasively and rapidly predict clarithromycin and fluoroquinolone resistance, thereby guiding personalized eradication therapies.

## Material and methods

### Ethical considerations

This study was approved by the Ethics Committee of Nanjing First Hospital (Approval Number: XQ202200516-01), The First Affiliated Hospital of Nanchang University(Approval Number: IRB-2022-177), Zhejiang Provincial Hospital of Chinese Medicine(Approval Number: 2022-Q-005-01), and Jiangsu Taizhou People’s Hospital(Approval Number: SJ 2023-008-02).

### Study design

1) This prospective, double-blind, multicenter, observational cross-sectional study was conducted across four tertiary hospitals in China (August 2022–October 2023). Eligible participants underwent simultaneous gastroscopy with gastric biopsy collection (for *H. pylori* culture and phenotypic susceptibility testing) and stool sampling (for fecal molecular resistance profiling by novel diagnostic kit and Sanger sequencing). The study protocol was registered at ClinicalTrials.gov (NCT05410652).

### Study population

Inclusion Criteria: 1) Adults aged 18–70 years with dyspeptic symptoms or confirmed *H. pylori* infection; 2) Willing to undergo gastroscopy and provide stool samples; 3) No antibiotic/bismuth use within 4 weeks or proton pump inhibitors (PPIs) within 2 weeks prior to enrollment. Exclusion Criteria: 1) Inadequate stool sample volume or unqualified biopsy specimens for culture; 2) Active gastrointestinal bleeding, pregnancy, or severe comorbidities (e.g., cirrhosis, renal failure); 3) Previous *H. pylori* eradication therapy within 6 months. Participant Flow: 1) Enrolled: 1,212 participants; 2) Excluded: 36 (2.97%) due to unqualified samples or protocol deviations. Final Cohort: 1,176 participants (327 from Nanjing, 385 from Nanchang, 222 from Hangzhou, 242 from Taizhou).

### Culture and antibiotic susceptibility test

A gastroscope (Olympus Corporation, Tokyo, Japan) was used to obtain gastric mucosal samples from study participants. For each patient, we obtained two gastric antrum mucosal biopsy specimens and one gastric body mucosal biopsy specimen. Gastric biopsy specimens were immediately placed in sterile transport medium (phosphate-buffered saline with 20% glycerol, pH 7.4) and stored at 4°C. Samples were transported to the microbiology laboratory within 2 hours post-collection. *H. pylori* was cultured on Columbia agar plates (Shanghai Kemajia Microbiotech Co., Ltd) supplemented with 5% sheep blood (Bio-Kont, Zhejiang, China). The agar was supplemented with vancomycin (1 mg/ml), polymyxin B (0.5 mg/ml), and amphotericin B (0.5 mg/ml) (Duly Biotech, Nanjing, China). to suppress the growth of other bacterial species. The plates were incubated in an environment containing 10% carbon dioxide and 5% oxygen, which is optimal for the microaerophilic growth of *H. pylori*. This incubation temperature was 37°C for 96 to 120 hours. *H. pylori* isolates were confirmed via Gram staining, urease activity, and *16S rRNA* sequencing. The phenotypic antibiotic resistance of *H. pylori* strains was assessed using the E-test method (Miftahussurur et al., 2020). A bacterial suspension standardized to 0.5 McFarland was inoculated onto culture plates, and MIC values were determined via gradient diffusion strips (E-test, bioMérieux, Marcy-l’Étoile, France) at the intersection of the inhibition zone with the strip. Phenotypic results were cross-validated against the ATCC 43504 reference strain to ensure species-level identification. The *H. pylori* ATCC 43504 reference strain used in this study was purchased by Beijing Baiou Bowei Biotechnology Co., Ltd (Beijing, China) and has been preserved in the Microbiology Laboratory of Nanjing First Hospital’s Department of Gastroenterology under standardized conditions. MIC values were interpreted using European Committee on Antimicrobial Susceptibility Testing (EUCAST) 2019 criteria to interpret MIC values for clarithromycin (resistance >0.25 µg/mL) and levofloxacin (resistance >1 µg/mL) (The European Committee on Antimicrobial Susceptibility Testing, 2019).

### Genomic DNA extraction, sequencing, and mutation analysis

Total genomic DNA was isolated from stool specimens using a commercial kit (Cowin Biosciences, Jiangsu, China). *H. pylori*-specific DNA was confirmed via *16S rRNA* PCR prior to resistance mutation analysis. Target amplification of *23S rRNA* and *gyrA* gene regions was achieved through PCR using specific primer pairs: *23S rRNA*_F (5′-ATGGGAGCTGTCTCAACCAGAG-3′) with *23S rRNA*_R (5′-CTCCATAAGAGCCAAAGCCCTTACT-3′) generating 177 bp products, and *gyrA*_F (5′-GATCGTGGGTGATGTGATTGGTA-3′) paired with *gyrA*_R (5′-GCCTTAGTCATTCTGGCTTCAGTG-3′) producing 186 bp amplicons. Purified PCR products underwent bidirectional Sanger sequencing through Sangon Biotech (Shanghai, China), with subsequent sequence alignment using DNAMAN software (v2005, Lynnon Biosoft) and heteroresistance evaluation via ContigExpress (v2000, InforMax) against the *H. pylori* 26695 reference genome. Resistance genotypes were defined through codon-level analysis: clarithromycin resistance required A2142C/G or A2143G mutations in *23S rRNA*, with heteroresistance indicating mixed wild-type/mutant sequences, while levofloxacin resistance criteria involved amino acid substitutions at *gyrA* positions 87 (Asn→other) and 91 (Asp→other), where heteroresistance denoted concurrent detection of both wild-type and mutant codons (Lauener et al., 2019; Malfertheiner et al., 2022).

### Melting curve-based Multiplex RT-PCR (gyrA/23S rRNA gene mutation diagnosis kit)

A Multiplex RT-PCR assay with melt curve analysis was developed to concurrently identify clarithromycin and levofloxacin resistance markers in *H. pylori*. The platform incorporated three detection systems: (1) a 192-bp *23S rRNA* target amplified by primers 5′-GCATGAATGGCGTAACGAGAT-3′ (forward) and 5′-ATAAGAGCCAAAGCCCTTACTTCAAAG-3′ (reverse), monitored via ROX-labeled probe 5′-GCAAGACGGAAAGACCCCGTG-3′ for A2142C/G and A2143G mutations; (2) an 85-bp *gyrA* fragment amplified using 5′-GATCGTGGGTGATGTGATTGGTA-3′ (forward) and 5′-AAAATCTTGCGCCATTCTCACTA-3′ (reverse) primers, coupled with a FAM-conjugated probe containing locked nucleic acid (LNA) modifications (5′-CGAT (CLSI, 2016) AT (Wang et al., 2018)GGTTTA (Beckman et al., 2017) GA[T]GC-3′) to detect codon 87 (*A260T/C261A/T261G*) and 91 (*G271A/T/A272G*) variants; (3) a 96-bp β-actin internal control amplified by 5′-CCATCCTGCGTCTGGACCT-3′ (forward) and 5′-CCGTGGTGGTGAAGCTGTAG-3′ (reverse) primers with Cy5-labeled probe verification. Thermal cycling involved initial denaturation (94°C/1 min), 55 amplification cycles (94°C/15 s → 60°C/30 s), followed by melt curve generation through gradual temperature ramping (0.02°C/s from 45°C to 75°C) after final strand separation (94°C/30 s) and primer annealing (45°C/60 s). Fluorescence signatures were analyzed across channels: Cy5 validation confirmed reaction integrity, while ROX/FAM Tm deviations ≥0.5°C indicated mutation profiles.

### Statistical analysis

Data analysis was performed using IBM SPSS 26.0 (Chicago, Illinois, USA). All the measured variables were tabulated using descriptive statistics. Sensitivity, specificity, and 95% confidence intervals (CIs) were computed using a 2 × 2 table to compare outcomes between the *gyrA/23S rRNA* gene mutation diagnostic kit and Culture, as well as between the kit and Sanger sequencing. The Kappa consistency test was used to assess the concordance between the detection results of the *gyrA/23S rRNA* gene mutation diagnostic kit method and the other two conventional testing techniques. In the realm of statistical analysis, the Kappa coefficient is bounded within the range of -1 to 1. Generally, a Kappa value exceeding 0.75 indicates a significant level of consistency.

## Results

### Features of subjects

**Figure 1:**
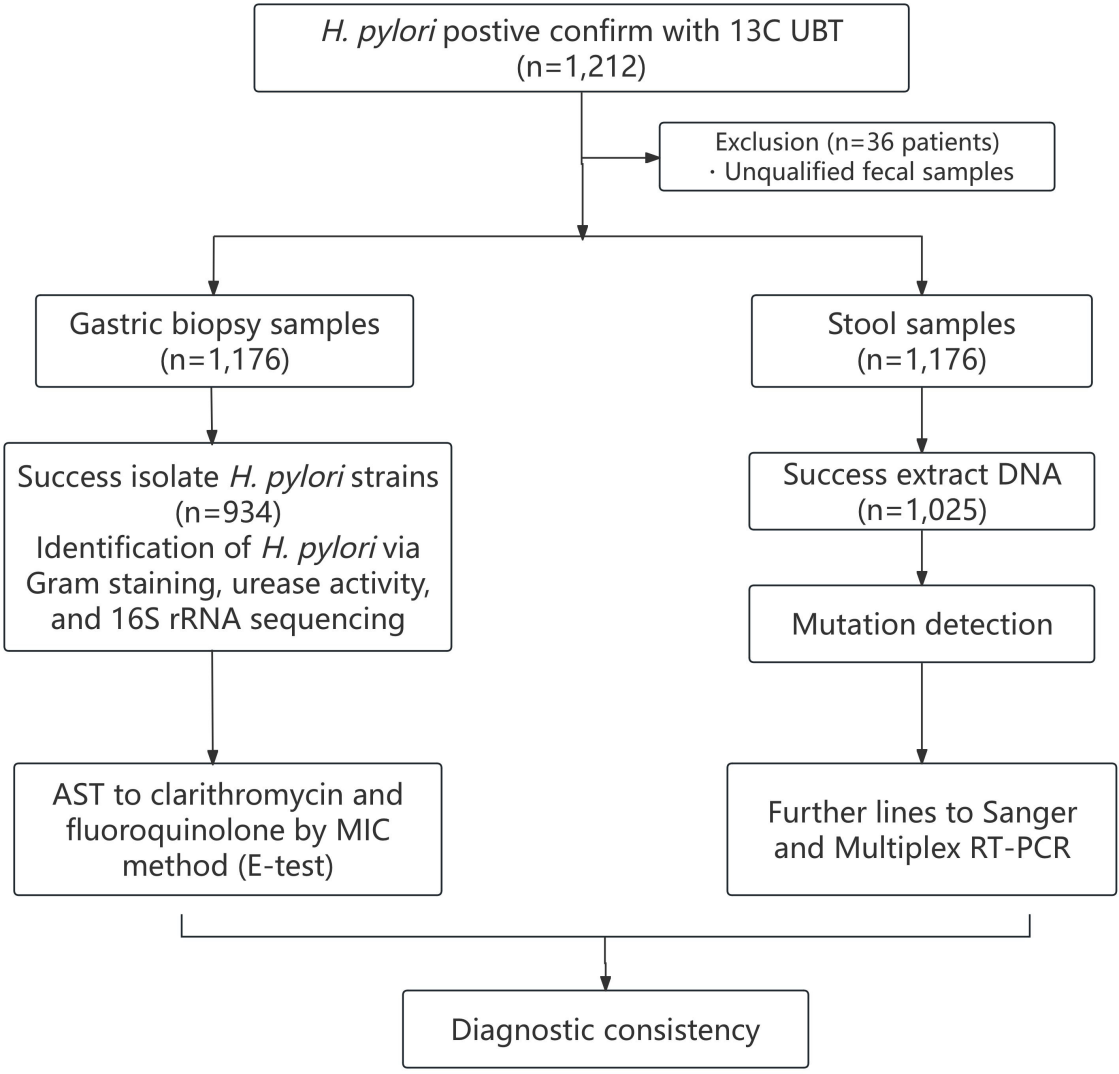
Flow chart of participants inclusion and analysis.

This study enrolled 1,212 participants, with 36 excluded for unqualified fecal samples. The 1,176 eligible participants underwent gastroscopy for gastric biopsy collection and stool sampling for fecal DNA extraction. Bacterial culture from gastric biopsies was successful in 934 (79.42%) cases, while DNA isolation from stool samples yielded 1,025 (87.16%) valid results. For levofloxacin phenotypic testing, however, another 24 strains were excluded due to ambiguous MIC endpoints during E-test interpretation. (910 valid samples) Likewise, for levofloxacin genotypic analysis, 83 samples excluded due to low *gyrA* amplification efficiency. (942 valid samples) These exclusions reflect adherence to standardized protocols to prioritize data reliability over sample quantity. Phenotypic resistance was determined via E-test (culture-based), and genotypic mutations were detected using the multiplex RT-PCR kit and sanger sequencing (stool-based). The number of effective participants from Nanjing First Hospital, The First Affiliated Hospital of Nanchang University, Zhejiang Provincial Hospital of Chinese Medicine, and Jiangsu Taizhou People’s Hospital were 327, 385, 222, and 242, respectively. **Figure 1** shows a flowchart illustrating the study.

**Table 1** describes demographic, clinical (symptoms and comorbidities), and endoscopic features of the 1,176 enrolled patients. Among all participants, male patients constituted 49.4%, whereas female patients comprised 50.6%. The ages of participants ranged from 13 to 19 years, yet the age group of 51 to 90 years demonstrates the highest proportion of patients within the studied population.

**Table 1:** Demographic and endoscopic characteristics of 1,176 participate.

Category	Feature	Number (n=1176)	Proportion (%)
Demographics	Sex		
	Male	581	49.40
	Female	595	50.60
	Age (years)		
	13-30	193	16.41
	31-50	452	38.44
	51-90	531	45.15
Clinical Features	Common Symptoms		
	*epigastric pain*	290	24.66
	acid reflux	160	13.61
	bloating	73	6.21
	Nausea	35	2.98
	Abnormal bowel movements	47	3.99
Endoscopic Features	Asymptomatic	571	48.55
	Lesion Type		
	Gastric angle/pyloric ulcer	65	5.53
	Esophagitis (LA-B or higher)	78	6.63
	Postoperative gastric changes	31	2.64
	Pseudodiverticulum	9	0.77
	Duodenal "frosted" ulcer	53	4.51
	Verrucous elevation/superficial depression	47	4.00
	Bile reflux	82	6.97
	Esophageal varices/cysts	12	1.02
	Isolated gastric mucosal erosion	215	18.28
	No obvious endoscopic lesions or Chronic gastritis	586	49.83

### Comparison of different methods for detecting clarithromycin resistance

The susceptibility of 934 *H. pylori* strains to clarithromycin was evaluated using the E-Test method. Consequently, 479 (51.28%) were susceptible to clarithromycin (MIC ≤ 0.25 mg/L), whereas 455 (48.72%) were resistant to clarithromycin (MIC > 0.25 mg/L). The diagnostic kit demonstrated high concordance with MIC-based phenotypic resistance. For clarithromycin resistance, the positive percentage agreement (PPA) was 92.97% (95% CI: 90.6–95.3%), negative percentage agreement (NPA) 87.89% (85.0–90.8%), and overall percentage agreement (OPA) 90.36% (88.5–92.3%) with a Cohen’s Kappa coefficient of 0.807 (**Table 2**).

**Table 2:** A comparison between different methods for detecting clarithromycin resistance.

Control method		Detection of clarithromycin resistance by fecal kits			PPA (%)	NPA (%)	OPA (%)	Kappa^b^	Asymp. Std. Error	Approx.Tb	Approx. Sig
		Positive	Negative	Total							
Culture	Positive	423	32	455	92.97 [90.6-95.3]^a^	87.89 [85.0-90.8]^a^	90.36 [88.5-92.3]^a^	0.807	0.019	24.714	0.000
	Negative	58	421	479							
	Total	481	453	934							
Sanger sequencing	Positive	508	15	523	97.13 [95.7-98.6]^a^	94.62 [92.6-96.6]^a^	95.90 [94.7-97.1]^a^	0.918	0.012	29.398	0.000
	Negative	27	475	502							
	Total	535	490	1,025							

PPA, Positive percentage agreement; NPA, Negative percentage agreement; OPA, Overall percentage agreement; R, resistant; S, Susceptible.

^a^95% Confidence Interval.

^b^Kappa: Poor agreement <0.20; Fair agreement 0.20-0.40.

A total of 1,025 fecal samples satisfied the testing criteria and underwent both Sanger sequencing and *gyrA/23S rRNA* fecal diagnostic kit analysis. The positive percentage agreement between the two methods was 97.13% (95% CI: 95.7% to 98.6%), the negative percentage agreement was 94.62% (95% CI: 92.6% to 96.6%), and the overall percentage agreement was 95.90% (95% CI: 94.7% to 97.1%). The Kappa value was 0.918 (**Table 2**).

**Supplementary Table S1** presents the concordance analysis between the fecal diagnostic kit and Sanger sequencing stratified by individual mutation types (e.g., A2142G, A2142C, A2143G in *23S rRNA*). Each mutation site was counted independently, resulting in higher total case numbers than the patient cohort size (n=1,025) due to samples harboring multiple resistance-associated mutations.

### Comparison of different methods for detecting levofloxacin resistance

The E-test results revealed that out of 910 *H. pylori* strains, 426 (46.81%) displayed resistances to levofloxacin, and 484 (53.19%) were sensitive. The *gyrA/23S rRNA* gene mutation diagnostic kit and E-Test method showed a positive percentage agreement of 86.85% (95% CI: 83.6% to 90.1%), a negative percentage agreement of 91.12% (95% CI: 88.6% to 93.7%), and an overall percentage agreement of 89.12% (95% CI: 87.1% to 91.1%). The Kappa coefficient was 0.781 (**Table 3**).

**Table 3:** A comparison between different methods for detecting Levofloxacin resistance.

Control method		Detection of levofloxacin resistance by fecal kits			PPA (%)	NPA (%)	OPA (%)	Kappa^b^	Asymp. Std. Error	Approx.Tb	Approx. Sig
		Positive	Negative	Total							
Culture	Positive	370	56	426	86.85 [83.6-90.1]^a^	91.12 [88.6-93.7]^a^	89.12 [87.1-91.1]^a^	0.781	0.021	23.573	0.000
	Negative	43	441	484							
	Total	413	497	910							
Sanger sequencing	Positive	400	14	414	96.92 [94.9-98.4]^a^	93.18 [91.0-95.3]^a^	94.69 [93.3-96.1]^a^	0.893	0.015	27.435	0.000
	Negative	36	492	528							
	Total	436	506	942							

PPA, Positive percentage agreement; NPA, Negative percentage agreement; OPA, Overall percentage agreement; R, resistant; S, Susceptible.

^a^95% Confidence Interval.

^b^Kappa: Poor agreement <0.20; Fair agreement 0.20-0.40.

Only 942 fecal samples were analyzed for *gyrA* gene mutations using Sanger sequencing and fecal diagnostic kits. In evaluating levofloxacin resistance, the positive percentage agreement between the *gyrA/23S rRNA* gene mutation diagnostic kit and the E-Test was 96.92% (95% CI: 94.9% to 98.4%). The negative percentage agreement was 93.18% (95% CI: 91.0% to 95.3%), whereas the overall percentage agreement was 94.69% (95% CI: 93.3% to 96.1%). The Kappa coefficient was 0.893 (**Table 3**).

**Figure 2:**
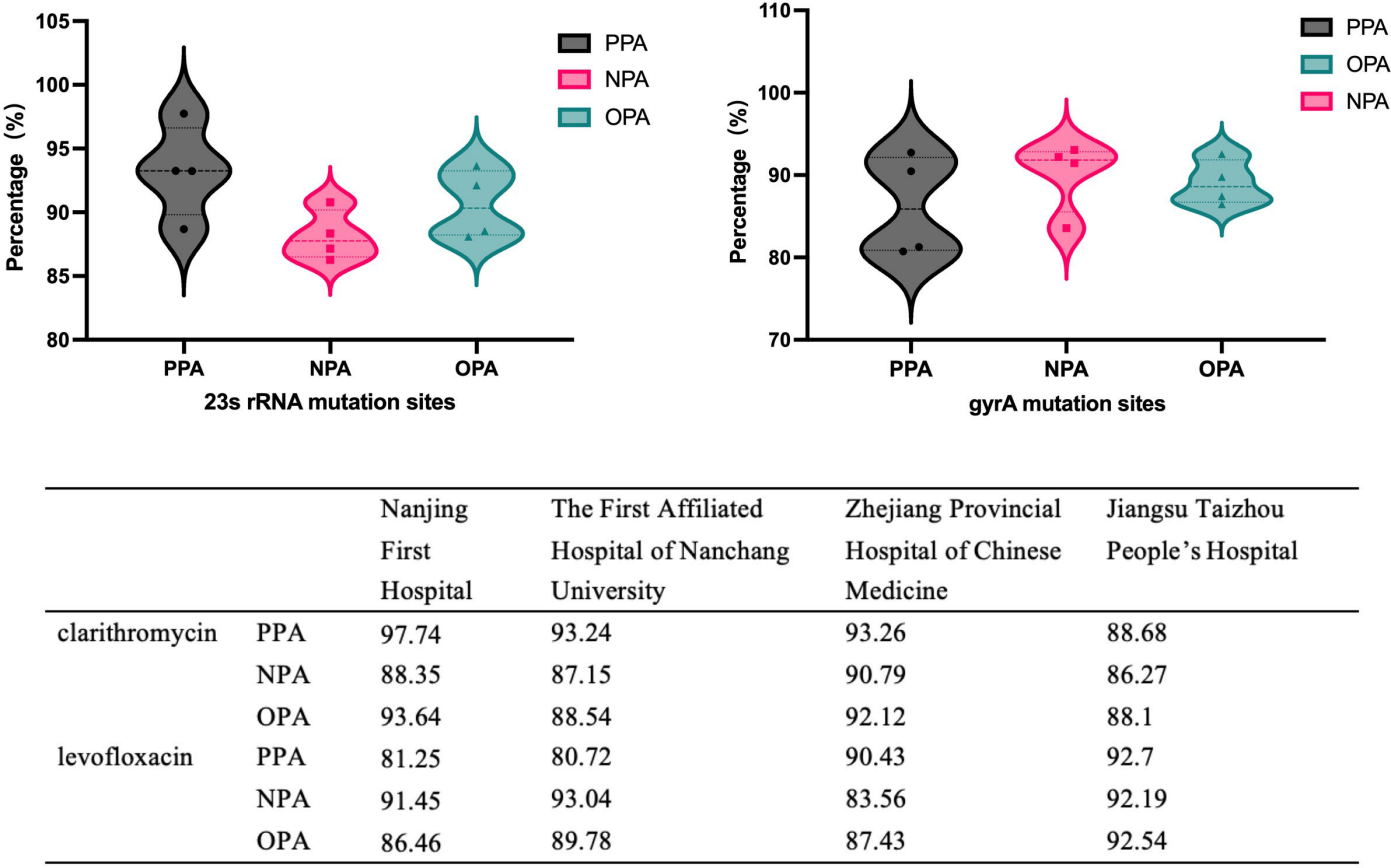
The concordance rate between the fecal drug resistance detection kit and the drug sensitivity test results across each center.

**Figure 2** shows the concordance rate between the fecal drug resistance detection kit and the drug sensitivity test results across each center. Nanjing First Hospital had a superior PPA in detecting clarithromycin resistance when using diagnostic kits and culture methods. However, Jiangsu Taizhou People’s Hospital had the highest PPA among the sub-centers for levofloxacin resistance testing. Data variations across each sub-center were relatively minor, with PPA, NPA, and OPA all exceeding 80%.

**Supplementary Table S2** evaluates the concordance between the fecal diagnostic kit and Sanger sequencing stratified by individual *gyrA* mutation types (N87K, D91N, D91Y, D91G). Each mutation site was analyzed independently, leading to a higher total case count (n=942) compared to the actual patient number due to samples harboring multiple resistance-associated mutations within the *gyrA* gene.

### The effect of *H. pylori* minimum inhibitory concentrations on the sensitivity of fecal diagnostic kits

**Figure 3:**
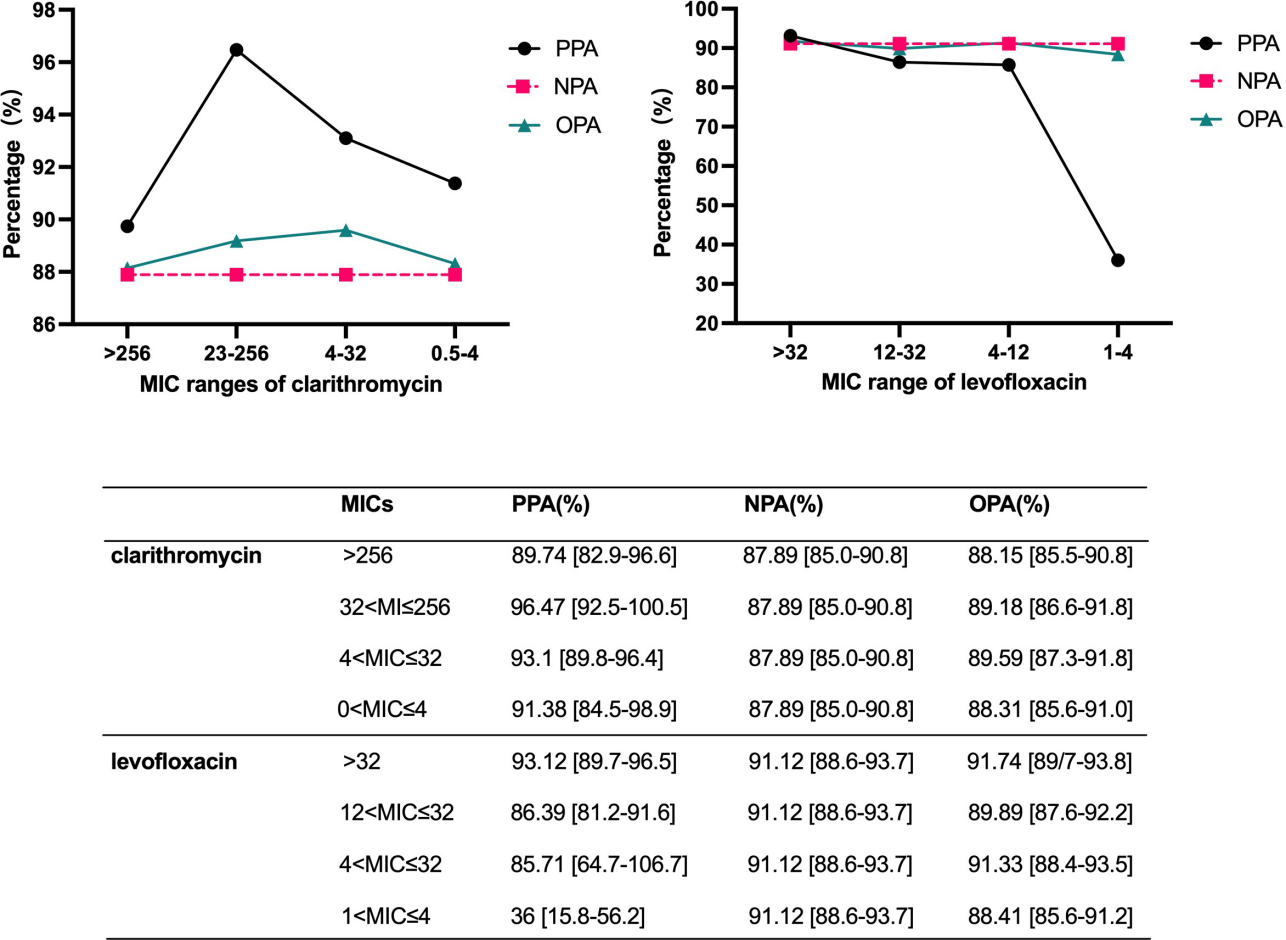
Assessment of the positive, negative, and overall percentage agreement of fecal kits in detecting strains with diverse MIC value.

A total of 455(48.72%) strains displayed resistance to clarithromycin based on the use of the E-Test technique. The MIC of the strains was categorized into four distinct intervals: MIC > 256, 32 < MIC ≤ 256, 4 < MIC ≤ 32, and 0 < MIC ≤ 4. **Table 4** shows the distribution of bacterial strains across each interval. **Figure 3** presents the PPA, NPA, and OPA between the results obtained from the fecal diagnostic kit and the *in vitro* culture susceptibility test. The PPA for clarithromycin resistance is relatively low compared to other groups when the MIC exceeds 256. The kit achieves its highest positive coincidence rate, at 96.47% when the MIC is greater than 32 but less than or equal to 256. However, the concordance between fecal detection kits and *in vitro* drug susceptibility assays in identifying clarithromycin resistance in *H. pylori* was maximized when the MIC ranged from 4 to 32 µg/mL. The Kappa coefficient was 0.774.

**Table 4:** Comparing the consistency between fecal kits and the E-test method in detecting clarithromycin resistance in strains with diverse MIC levels.

Control method			Detection of clarithromycin resistance by fecal kits			Kappa value^a^	Asymp. Std. Error	Approx. Tb	Approx. Sig
			Positive	Negative	Total				
Culture	Positive	>256	70	8	78	0.612	0.042	15.114	0.000
		32<MIC ≤ 256	82	3	85	0.666	0.038	16.593	0.000
		4<MIC ≤ 32	216	16	232	0.774	0.025	20.806	0.000
		0<MIC ≤ 4	55	5	60	0.574	0.046	14.27	0.000
	Negative	MIC ≤ 0.5	58	421	479				
	Total		481	453	934				

PPA, Positive percentage agreement; NPA, Negative percentage agreement; OPA, Overall percentage agreement; R, resistant; S, Susceptible.

^a^Kappa: Poor agreement <0.20; Fair agreement 0.20-0.40.

For levofloxacin, the bacterial strains were categorized into four distinct intervals based on their MIC values: MIC > 32, 12 < MIC ≤ 32, 4 < MIC ≤ 12, and 1 < MIC ≤ 4. In the analysis of levofloxacin resistance, the PPA markedly decreased to 36.00%when the MIC was ≤ 4 mg/L. Conversely, concordance significantly improves as the MIC increases. The concordance between the diagnostic kit results and the drug susceptibility test outcomes is maximized when the MIC of levofloxacin exceeds 32, yielding a Kappa coefficient of 0.814. However, the sample size with a MIC ≤ 4 mg/L is limited to 25, representing only 5.87% of the total drug-resistant cases. This relatively small proportion minimally influences the overall diagnostic outcomes.

## Discussion

The antibiotic resistance of *H. pylori*, specifically to clarithromycin, levofloxacin, and metronidazole significantly contributes to treatment failure. Of note, phenotypic methods are commonly employed to identify *in vitro* drug resistance. However, these methods require the cultivation of bacterial strains (Vega et al., 2007). Several research results have shown a high concordance between resistance gene mutations and drug sensitivity outcomes for clarithromycin and levofloxacin (Zhong et al., 2021; Jiang et al., 2022; Wei et al., 2023). This underpins the theoretical foundation for developing gene detection kits, which could promote the diagnosis of *H. pylori* resistance to these antibiotics.

Several commercial kits have been developed for detecting *H. pylori* resistance mutations outside China. The GenoType HelicoDR kit (Hain Lifescience, Germany) utilizes reverse hybridization to detect clarithromycin (*23S rRNA*, A2142G/C/A2143G) and fluoroquinolone (*gyrA*, N87K/D91N/Y/G) resistance mutations from gastric biopsies. A multicenter study reported 94.3% sensitivity, and 98.5% specificity compared to phenotypic testing, but its reliance on invasive biopsies limits clinical utility (Pastukh et al., 2017). In Europe, the ClariRes Assay (Ingenetix, Austria) enables non-invasive detection of clarithromycin resistance (*23S rRNA* mutations) directly from stool samples, achieving 95% concordance with E-Test results (Beckman et al., 2017). However, it cannot simultaneously assess fluoroquinolone resistance. Similarly, a Japanese LAMP-based POCT kit (LSI Medience Corporation) detects *H. pylori* and clarithromycin resistance (A2143G) from gastric juice with 100% sensitivity and 95.9% specificity yet requires gastric fluid sampling and lacks multiplex capability (Tsuda et al., 2022).

The non-invasive nature of fecal resistance detection holds transformative potential for clinical practice. Unlike endoscopy-dependent methods, stool-based PCR enables widespread screening, real-time monitoring of resistance trends, and personalized therapy without procedural risks—a priority underscored in recent advancements (Celiberto et al., 2023). By rapidly identifying clarithromycin- and fluoroquinolone-resistant strains, this approach empowers clinicians to bypass ineffective empiric regimens, reducing treatment failures and secondary resistance development.

To our knowledge, three types of gene mutation detection kits have been subjected to clinical studies in China so far; however, all these kits have been assessed using gastric mucosal samples. The three types of detection kits include: (1) the *23S rRNA* gene and *gyrA* gene mutation detection kit developed by Jiangsu Mole Bioscience Co., Ltd.; (2) the *23S rRNA* gene mutation detection kit developed by Shanghai Outdo Biotech Co., Ltd.; and (3) the *23S rRNA* mutation detection kit developed by Youlian Ruikang (Shanghai) Gene Technology Co., Ltd. The first two types of diagnostic kits have obtained Class III medical device registration certificates, approved by the National Medical Products Administration (NMPA). Nevertheless, the use of these three diagnostic test kits for detecting drug resistance requires that patients undergo gastroscopy to obtain gastric mucosal samples, hence significantly constraining their broader application.

The present study sought to assess the clinical efficacy of China’s first *H. pylori* fecal molecular resistance diagnostic kit i.e., a fecal *gyrA/23S rRNA* gene mutation diagnostic kit. We compared three techniques for detecting mutations in the *gyrA* and *23S rRNA* genes, i.e., (1) novel fecal diagnostic kit; (2) culture methods; and (3) Sanger sequencing. The four sub-centers participating in this study are located in four cities across three distinct provinces in China. The analysis from each sub-center showed no statistically significant regional variation in positive, negative, and overall percentage agreement between the results from the reagent kit and the culture method.

Our findings demonstrated a good concordance between the fecal diagnostic kit and both the E-test method and direct sequencing. For clarithromycin, the PPA and NPA between the fecal diagnostic kit and E-test were 92.97% and 87.89%. Studies have shown that the *H. pylori* ClariRes Assay (Ingenetix) has sensitivity and specificity values of 83%-98% and 98%-100%, respectively, for detecting clarithromycin resistance in stool samples (Vecsei et al., 2010; Scaletsky et al., 2011). The *23S rRNA* gene and *gyrA* gene mutation detection kit developed by Jiangsu Mole Bioscience Co., Ltd. has a clinical sensitivity of 91.81% and specificity of 80.33% for detecting clarithromycin resistance from the gastric samples, respectively. Th*e 23S rRNA* gene mutation detection kit, developed by Shanghai Outdo Biotech Co., Ltd., boasts a clinical sensitivity of 94.60% and a specificity of 87.89% in detecting clarithromycin resistance. Another domestic study revealed that the concordance rate between the detection of the *23S rRNA* gene using a gastric mucosa detection kit and conventional drug sensitivity testing was 90.44% (Zhang et al., 2018). The fecal diagnostic kit for detecting clarithromycin resistance demonstrated a positive percentage agreement similar to the findings from foreign research, at the same time also exhibiting similar sensitivity and specificity to the existing gastric mucosa detection kit available in China.

Previous large-scale studies have shown a concordance rate of 89.12% between gastric mucosal PCR sequencing outcomes and fluoroquinolone antibiotic susceptibility test results (Zhong et al., 2021). The *23S rRNA* gene and *gyrA* gene mutation detection kit developed by Jiangsu Mole Bioscience Co., Ltd. exhibits a clinical sensitivity of 84.96% and specificity of 82.16% for detecting fluoroquinolone antibiotic resistance, respectively. Clinical research results in China have shown that *gyrA* mutation detection kits for gastric mucosa have an 81.62% consistency with drug susceptibility testing (Zhang et al., 2018). The present study used fecal samples. The PPA and NPA between the fecal detection kit and the drug sensitivity test reached 86.85% and 91.12%, respectively, yielding an overall agreement rate of 89.12%. These findings represent an improvement, unlike the previously developed diagnostic kits.

This study also confirmed that the MIC value of the *H. pylori* strain influences the PPA and NPA of fecal detection kits. For clarithromycin, the PPA tends to increase as the MIC value increases when the MIC value ranges from 0 to 256 mg/L. However, the PPA begins to decline once the MIC value surpasses 256 mg/L. For levofloxacin, the positive agreement rate also increases as the MIC value increases within the range of 0 to 32 mg/L. The aforementioned research findings can provide more comprehensive insights into the clinical application of this kit and better guidance for clinical medication decisions.

This work observed discrepancies between the results of fecal kits and drug sensitivity tests. The false positives of the fecal kit can be attributed to two primary reasons: first, the strain may revert to its sensitive phenotype due to various other factors; secondly, some samples may contain a mixture of heteroresistance strains. Generally, sensitive strains in the microbial flora grow more rapidly than drug-resistant strains, causing drug sensitivity test results that predominantly show the phenotype of the sensitive strains. Heteroresistance poses a challenge for phenotypic methods like culture, which often fail to detect minority resistant subpopulations due to their slower growth rates under selective pressure. In contrast, molecular methods (e.g., multiplex RT-PCR or sequencing) can identify low-frequency resistance-associated mutations in stool samples, even when resistant strains are outcompeted *in vitro*. This phenomenon may explain instances where the fecal kit detected resistance mutations (e.g., A2143G in *23S rRNA*) while culture results indicated susceptibility. The underlying factors causing false negative outcomes in stool diagnostic kits can be traced to the following two reasons: (1) Certain strains have uncommon drug resistance mutation sites that cause a drug-resistant phenotype; however, these mutations may not be detected by the kit; (2) In some individual stool samples, *H. pylori* concentration was too low for the fecal kit to detect any drug resistance mutations. Similarly, inconsistencies can be found between the results of the fecal kit and sequencing. The fecal kit may yield false positive results when additional mutation sites are present in the sequence at the detection probe of the fecal reagent. For certain low-frequency mutations, the detection threshold of the fecal test kit is higher unlike that of sequencing, causing false negative results in the test kit. causing false negative results in the test kit.

Notably, the Maastricht VI/Florence Consensus Report emphasized the necessity of early resistance-guided therapy in regions with high clarithromycin (>20%) and fluoroquinolone (>15%) resistance rates, as empiric regimens risk therapeutic failure. The non-invasive fecal diagnostic kit evaluated here supports this recommendation by enabling rapid resistance profiling. The diagnostic kit can qualitatively detect specific mutations including A2142G, A2142C, and A2143G in the *23S rRNA* gene, alongside N87I/K and D91N/Y/G mutations in the *gyrA* gene. These mutation sites are responsible for *H. pylori* resistance to clarithromycin and fluoroquinolones. Furthermore, the kit shows a high PPA and NPA in the auxiliary diagnosis of *H. pylori* resistance, effectively fulfilling the requirements of clinical practice.

However, this work still has its limitations. First, the *gyrA/23S rRNA* gene mutation diagnosis kit is designed for qualitative diagnosis and cannot distinguish specific types of mutation sites during testing. Secondly, we failed to obtain an adequate number of mutant strains with the rare site mutation (2142C) for testing purposes. However, we will continue collecting mutant strains with the 2142C mutation and use fecal assay kits for further testing to validate the sensitivity of the diagnostic kit in diagnosing this rare mutation. Beside this, we acknowledge that cultured isolates were not subjected to molecular characterization of resistance determinants (e.g., *23S rRNA* or *gyrA* sequencing) to directly compare with stool-based results. This limitation stems from resource constraints and the study’s primary focus on validating clinical utility (kit vs. phenotypic susceptibility). Prior studies have shown strong concordance between stool PCR and isolate sequencing for resistance mutations (>95% agreement), suggesting minimal discordance in real-world settings (Beckman et al., 2017). Future work will integrate isolate sequencing to refine heteroresistance detection thresholds.

## Data Availability

The raw data supporting the conclusions of this article will be made available by the authors, without undue reservation.
