# Supplementary material for: Clinical evaluation of a novel H. pylori fecal molecular diagnosis kit (multiplex RT-PCR method) for detecting clarithromycin and fluoroquinolones resistance using stool samples
Source: Front Cell Infect Microbiol. 2025 Jun 23;15:1592612. doi: 10.3389/fcimb.2025.1592612 (PMC12230099; doi:10.3389/fcimb.2025.1592612)
Supplement: Supplementary file 1 [file Table1.docx]

**Table S1. Concordance analysis between Sanger sequencing results and fecal diagnostic kit detection for *23S rRNA* mutation.**

| **Mutation Type** | ***gyrA/23S rRNA* fecal diagnostic kits** | **A2142G** | **A2142C** | **A2143G** | **Wild-Type** | **Total** |
| --- | --- | --- | --- | --- | --- | --- |
| **Sanger sequencing** | Positive | 23 | 9 | 489 | 27 | 548 |
|  | Negative | 0 | 1 | 15 | 475 | 492 |
|  | Total | 24 | 10 | 504 | 502 | 1,040^a^ |
| **Performance Metrics** | PPA (%) | 95.83 | 90 | 97.02 | - | - |
|  | NPA (%) | 94.62 | 94.62 | 94.62 | - | - |
|  | OPA (%) | 94.68 | 94.53 | 95.83 | - | - |

PPA: Positive percentage agreement；NPA: Negative percentage agreement; OPA: Overall percentage agreement;

a: The discrepancy in total case numbers arises because the fecal resistance detection kit is a qualitative assay that cannot distinguish between specific mutation types. In contrast, Sanger sequencing may reveal multiple co-occurring mutations within *23S rRNA* (e.g., both A2142G and A2143G). For statistical analysis, such cases were counted per mutation, resulting in higher aggregate numbers than the per-patient counts (1,025 for clarithromycin) mentioned in the main text.
