# Supplementary material for: Clinical evaluation of a novel H. pylori fecal molecular diagnosis kit (multiplex RT-PCR method) for detecting clarithromycin and fluoroquinolones resistance using stool samples
Source: Front Cell Infect Microbiol. 2025 Jun 23;15:1592612. doi: 10.3389/fcimb.2025.1592612 (PMC12230099; doi:10.3389/fcimb.2025.1592612)
Supplement: Supplementary file 2 [file Table2.docx]

**Table S2. Concordance analysis between Sanger sequencing results and fecal diagnostic kit detection for *gyrA* mutation.**

| **Mutation Type** | ***gyrA/23S rRNA* fecal diagnostic kits** | **N87I (A260T)** | **N87K (C261A)** | **N87K (T261G)** | **D91N (G271A)** | **D91Y (G271T)** | **D91G (A272G)** | **Wild-Type** | **Total** |
| --- | --- | --- | --- | --- | --- | --- | --- | --- | --- |
| **Sanger sequencing** | Positive | 29 | 152 | 82 | 95 | 40 | 77 | 36 | 511 |
|  | Negative | 0 | 7 | 1 | 5 | 1 | 0 | 492 | 506 |
|  | Total | 29 | 159 | 83 | 100 | 41 | 77 | 528 | 1,017^a^ |
| **Performance Metrics** | PPA (%) | 100.0 | 95.60 | 98.80 | 95.00 | 97.56 | 100 | - | - |
|  | NPA (%) | 93.18 | 93.18 | 93.18 | 93.18 | 93.18 | 93.18 | - | - |
|  | OPA (%) | 93.54 | 93.74 | 93.94 | 93.47 | 93.50 | 94.05 | - | - |

PPA: Positive percentage agreement；NPA: Negative percentage agreement; OPA: Overall percentage agreement;

a: The discrepancy in total case numbers arises because the fecal resistance detection kit is a qualitative assay that cannot distinguish between specific mutation types. In contrast, Sanger sequencing may reveal multiple co-occurring mutations within *gyrA* (e.g., N87K and D91N). For statistical analysis, such cases were counted per mutation, resulting in higher aggregate numbers than the per-patient counts (942 for levofloxacin) mentioned in the main text.
